# An analysis of the cost of postgraduate training in surgery in Ireland compared to other specialties

**DOI:** 10.1007/s11845-021-02641-z

**Published:** 2021-05-29

**Authors:** Earley H., Mealy K.

**Affiliations:** 1 Department of General Surgery, Wexford General Hospital, Newton Road, Wexford, Ireland; 2 Royal College of Surgeons, 123 St Stephen’s Green, Dublin, Ireland

**Keywords:** Cost of training, Funding, Healthcare economics, Speciality training, Surgery, Surgical training

## Abstract

**Introduction:**

Postgraduate specialty training in Ireland is associated with considerable cost. Some of these are mandatory costs such as medical council fees, while others are necessary to ensure career progression, such as attendance at courses and conferences. In particular, surgical specialities are believed to be associated with high training costs. It is unknown how these costs compare to those borne by counterparts in other specialities.

**Aims:**

The aims of this study were to

1. Quantify the amount that trainees in Ireland spend on postgraduate training
2. Determine whether a difference exists between surgery and other non-skill-based specialties in terms of expenditure on training

**Methods:**

A standardised non-mandatory questionnaire was circulated to trainees across two training centres in Ireland. Trainees at all levels were invited to participate.

**Results:**

Sixty responses were obtained. Fifty-seven questionnaires were fully completed and included for analysis. The median expenditure on training was higher for surgical than non-surgical specialities. Subgroup analysis revealed surgical training was associated with higher expenditure on higher degrees and courses compared to medical training (*p* = 0.035). > 95% of trainees surveyed felt that greater financial support should be available for trainees during the course of their training.

**Conclusions:**

This study demonstrated that a career in surgery is associated with higher ongoing costs for higher degrees and courses than counterparts in non-surgical training. All surgical trainees surveyed felt that better financial support should be available. Increasing financial support for may be a tangible way to mitigate against attrition during training.

## Introduction

In recent years, application numbers to surgical training have declined in many countries, including in Ireland [1–3]. Amongst reasons such as lack of medical student engagement, poor work-life balance and long duration of training, one of the reasons cited for the decline in interest in surgery as a career is the perceived cost of specialty training associated with surgery [4–7].

A large cross-sectional study from the Association of Surgeons in Training (ASiT) based in the UK and Ireland, published in 2017, found that surgical trainees gain much of their mandatory training at a significant personal cost [8]. This study also demonstrated that medical students are graduating with more debt than previously, when compared to a similar study conducted ten years earlier. The observation is likely due to a combination of factors including rising costs of University fees [9] and higher costs of living [10], as well as a higher number of students pursuing the Graduate Entry Programme into medicine. Evidence from the UK and USA suggests that student debt on graduation also impacts on speciality choice [11] and that it represents a significant financial burden during training [12].

Funding available to trainees to reimburse costs sustained during training is not in line with increasing costs of training, and indeed in Ireland in the time period from 2008 to 2018 had declined, adding to the financial burden of non-consultant hospital doctors (NCHDs) in training. In 2019, the Health Service Executive (HSE) Training Support Scheme fund increased to €1250 per anum for Senior House Officers (SHOs) and €2000 per anum for Specialist Registrars (SpRs), marking an improvement in the situation [13]. However, prior to 2019 the Clinical Course and Examination Refund Scheme covered only part of the membership examinations deemed mandatory for junior trainees [13].

In order to maintain a strong surgical work force in the future, it is important to understand the barriers and factors potentially deterring medical graduates from pursuing a career in surgery. Traditionally, the cost of skill-based specialties such as surgery was thought to be associated with higher training costs and data from the UK supports this. To date, no study has examined the cost of surgical training compared to other specialties in the Irish setting.

## Aims

The aims of this study were to quantify the amount of taxed income that trainees in Ireland spend on postgraduate training for surgical and non-surgical specialties. Secondary aims were to determine the different sources of training related expenditure and finally to determine whether a difference existed between surgical specialties and non-surgical specialties in terms of expenditure on training.

## Methods

### Questionnaire design

**Fig. 1:**
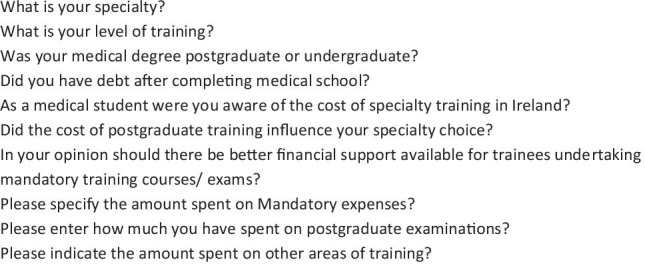
Ten-point questionnaire

A ten-point questionnaire was designed consisting of binominal and free text responses. The online platform SurveyMonkey (Palo Alto, California, USA; www.surveymonkey.com) was used to build and distribute the questionnaire in one hospital site. Hard copies of the survey were distributed in the second site, and data entered manually into the SurveyMonkey database. All individual question items were compulsory. No individually identifiable information was collected. No incentives were offered for participation. A link to the online survey was distributed to the mailing lists of all hospital-based trainees. Participants consented to the use of the analysis, distribution and publication of anonymised data. The questions included in the survey can be found in Fig. 1.

For analysis, training associated costs were broken down into mandatory, exams and other costs. Mandatory costs included medical council fees, registration with a professional body or the cost of clinical indemnity. Exams included membership examinations for the respective Royal Colleges, USMLE exams or fellowship examinations. The other category included costs associated with higher degrees, courses, attendance at conferences and meetings and the travel expenses associated with these activities. While many of these expenses are not deemed mandatory, many of them are a prerequisite to gain the necessary skills and training required and also to ensure advancement within a trainees’ chosen speciality.

What is your specialty?

What is your level of training?

Was your medical degree postgraduate or undergraduate?

Did you have debt after completing medical school?

As a medical student were you aware of the cost of specialty training in Ireland?

Did the cost of postgraduate training influence your specialty choice?

In your opinion should there be better financial support available for trainees undertaking mandatory training courses/ exams?

Please specify the amount spent on Mandatory expenses?

Please enter how much you have spent on postgraduate examinations?

Please indicate the amount spent on other areas of training?

## Participants

The study was distributed to NCHDs at all levels of training including interns, Senior House Officers (SHO), registrars and specialist registrars (SpRs) across the two clinical sites. These clinical sites included one University Teaching Hospital and one General Hospital. Inclusion of both model 3 and model 4 hospital settings aimed to ensure results that were representative of the non-consultant hospital doctor population in Ireland as a whole and to avoid bias associated with any one hospital site. Responses were obtained by open invitation and the pool size from which respondents were drawn comprised approximately 370 NCHDs.

## Data analysis

Data were graphed and analysed in Excel (Microsoft®, Redmond, Washington, USA). Statistical analysis was performed using SPSS software (SPSS Statistics, IBM, London, UK). Significance testing was conducted using the Mann-Whitney U test; statistical significance was accepted at *p* < 0.05.

## Results

### Cohort demographics

**Fig. 2:**
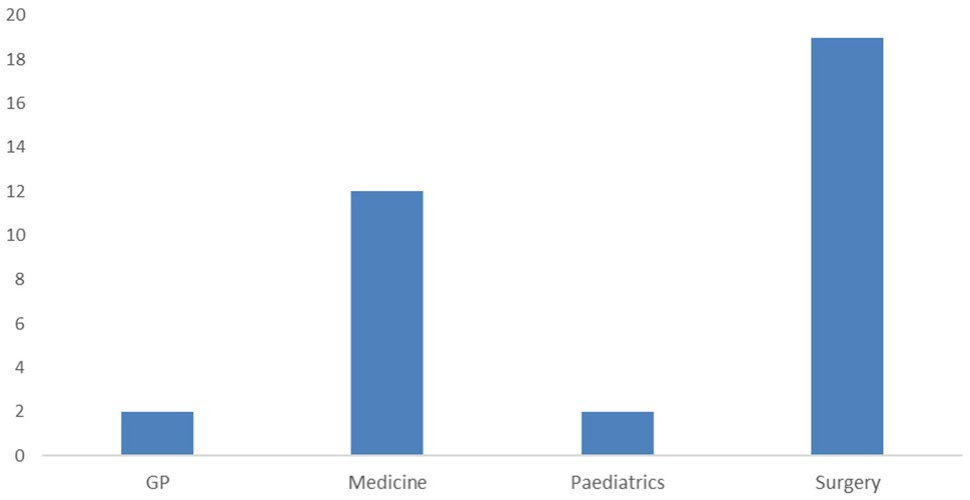
Breakdown of responses for those in specialty training by speciality

Sixty responses were obtained. Information on speciality or level of training was omitted in the case of 3 surveys, and these were omitted from further analysis. Fifty-seven responses were included for analysis. 37.3% of responses were from interns, 32% were SHOs and 30.5% were registrar or specialist registrar level of training. Of those in specialist training (SHOs, registrars and SpRs, *n* = 35) analysed, 19 responses (54.3%) were from surgical specialties (all surgical specialties were grouped as “surgery”) and 16 (45.7%) from non-surgical specialties (including medicine, paediatrics and those in general practice completing their hospital-based rotations). A breakdown of responses by individual speciality is outlined in Fig. 2.

58.3% of the total cohort had completed an undergraduate medical degree while 41.7% had completed a postgraduate medical degree. Sixty percent of those surveyed reported having debt on completion of medical school. 81.7% of those surveyed reported being unaware of the cost associated with postgraduate speciality training in Ireland, with 10% stating the potential cost of training influenced their choice of speciality. All those surveyed except one individual from the non-surgical cohort felt that better financial support should be available to trainees to support their training efforts.

## Costs associated with training

Cumulative costs incurred for the group of trainees as a whole (both surgical and non-surgical specialties), at the time of taking the survey, were evaluated first based on level of training. Median overall costs attained by the whole group and based on level of training are outlined in Table 1.

**Table 1:** Median expenditure by level of training (values expressed in €)

Median expenditure in euro (€)	Total	Mandatory	Exams	Other
Whole group	6100	870	1900	3600
Training Level				
Intern	4685	480	1700	2300
SHO	5120	1135	2150	2150
Registrar	15,200	1555	3750	8800

The median overall costs attained at intern level were in excess of €4000. The majority of these costs were mandatory expenses such as medical council registration, with a median expenditure of €480 reported for the “mandatory expenses” category for interns. Membership exams accounted for a large proportion of intern spending also. The median overall costs attained at SHO level was €5120. Exams and “other expenses” accounted for the majority of expenditure at SHO level. The median overall costs attained at registrar level were €15,200. A summary of overall median reported costs and median costs for each category is outlined in Table 1.

## Comparison of the cost of training between surgical and non-surgical specialities

**Fig. 3:**
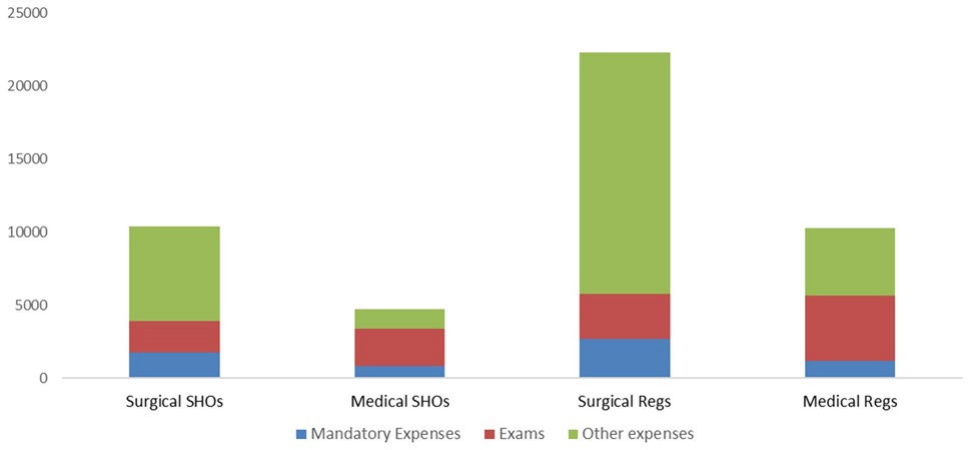
Median expenditure in each category based on speciality and level of training

For comparison of the costs associated with training between surgical vs non-surgical specialities, interns were omitted from analysis and analysis performed on data obtained from NCHDs at SHO, registrar or SpR level. Median expenses for each level of training by category are outlined in Fig. 3. Total median costs occurred during training were higher in surgery. However, on statistical analysis, this difference did not reach significance when analysed using the Mann-Whitney U test (*p* = 0.208). This held true when separate analysis was performed at both SHO (*p* = 0.211) and registrar level (*p* = 0.277).

Subgroup analysis was performed to assess differences in expenditure between surgical and non-surgical specialities for the groups “mandatory expenses”, “exams” and “other expenses”. This demonstrated that surgical training was associated with significantly higher expenditure in the “other expenses” category (courses, higher degrees, conferences, travel etc.), *p* = 0.035 (Fig. 3). Within this category, higher degrees accounted for the highest source of expenditure, followed by courses. 47.4% (9/19) of surgical trainees had completed a higher degree, compared to 12.5% (2/16) of non-surgical trainees. Medical training, on the other hand, was associated with higher median expenditure on the “exams” category (Fig. 3); however, this difference did not reach statistical significance (*p* = 0.541). No difference was noted between surgical and non-surgical groups in spending on the “mandatory expenses” category (*p* = 0.165).

## Discussion

In keeping with previously published literature [8], this study confirms that trainees across all specialties accumulate considerable costs over the course of their postgraduate training. Some are necessary expenses including registration with the medical council and training bodies. Others, such as courses and exams, are necessary to ensure career progression. This study highlights that costs of thousands of euro are associated with undertaking higher degrees, courses and the travel expenses associated with such endeavours over the course of training.

Although this study did not obtain information on how much of these expenses were refunded, the Clinical Course and Examination Refund Scheme (CCERS) offered by the HSE was 450 euro per membership examination or for a list of pre-approved courses prior to July 2019, when many of the participants in the current study completed their examinations [13]. This does not cover the full cost of the postgraduate exams for any specialties as demonstrated from this study and can only be claimed once per exam. Several trainees will require more than one attempt to pass at least one part of these examinations. In terms of other funding available, this exists in the form of the HSE Training Support Scheme (TSS) and the Specialist Training Fund, offered by training bodies to higher specialist trainees. The TSS has been increased in recent years and represents an improvement in financial support for trainees [13]. Notwithstanding this, based on the figures reported here, neither of these sources provides full support, and the majority of the cost of training is still conferred to the trainees. In particular, financial support for fees incurred for higher degrees is lacking.

Sixty percent of those surveyed reported having debt on completion of medical school, a statistic that is likely to rise, owing to increasing numbers of students entering Graduate entry medical programmes and increasing costs of tuition. Based on previous data, those who have accumulated significant debt may be less likely to pursue a specialty associated with high training costs [11, 12]. Interestingly, the cost of training did not influence the career choice of the majority of those who complete the present study. This may have been due in part, to the fact that the majority (> 80%) were unaware of the costs associated with training after completion of their undergraduate degree. A further point worth noting is that those undertaking a medical degree are more likely to be from affluent backgrounds, based on data from the Higher Education Authority [14]. The considerable financial barriers demonstrated in this survey may act as a deterrent to those of limited means from pursuing a career in surgery, or indeed any medical speciality. This study, by definition, excludes those who opted against a career in medicine from the outset, due to the perceived associated costs.

This study did, however, highlights the higher costs associated with surgical training. Although this did not reach significance on statistical analysis, it is nonetheless an important finding. The data also demonstrate that trainee surgeons have higher ongoing costs associated in particular with courses and higher degrees on subgroup analysis, a statistic that did reach significance. Surgery is becoming subspecialised, and trainees are required to attain an ever more specialist skill set. This, combined with reduced training time in light of run-through training schemes and the European Working Time Directive, has made it increasingly difficult to attain the necessary skill set in the clinical setting. To address this, it is necessary for trainees to gain skills at courses. In particular, simulation-based courses have been shown to enhance attainment of operative skill, particularly in areas such as laparoscopy [15] and robotic surgery [16, 17], and have also become popular to gain non-technical skills in high fidelity scenarios such as trauma [18]. These courses, as they become increasingly advanced, also increase in cost. A recent London-based study found that 89% of participants cited cost as a significant barrier to implementing surgical simulation-based programmes [19]. If there is to be a move towards more simulation-based training in the future, in order to ensure surgical training is attractive and sustainable, it must be associated with equitable training cost to the individual trainee.

Taking time out of training to undertake a higher degree is common place and a further source of cost among trainee surgeons [20]. Traditionally, a high publication rate was required to attain higher specialist training [21]. Data from this study indicate that, although there has been a move to run-through training schemes, a significant proportion of surgical trainees still undertake a higher degree. A further point worth considering, which was beyond the scope of the present study, is the cost associated with fellowship training. Most surgical trainees will complete at least 1 year in fellowship training, often undertaken abroad, a further factor associated with considerable personal cost.

A recent survey conducted amongst medical students in Ireland found that only 36% of those surveyed were considering a career in surgery [5]. Taking into account the attrition rate associated with the early years of surgical training [22], this is an alarming statistic for the future of surgery in Ireland. In order to maintain a competent work force in surgery, it is clear efforts must be made to mitigate factors acting as deterrents to graduates pursuing surgery as a career.

## Conclusion

This study demonstrated that a career in surgery is associated with higher ongoing costs for higher degrees and courses than counterparts in non-surgical training. All surgical trainees surveyed felt that better financial support should be available to trainees. A reduction in applications, combined with a high attrition rate in the early years of surgical training, poses a challenge to general surgery as a speciality [22, 23]. Increasing financial support for surgical trainees may represent a tangible way to mitigate against attrition during training.

## Limitations

The authors acknowledge that the present study has some limitations. The study relies on self-reported data which may be subject to recall bias in reporting of costs. This, however, would be difficult to control for on subsequent studies, and the reported costs were verified, where possible, based on the current cost of postgraduate exams and courses. A second limitation of this study is that a high percentage of those responding to the survey were at intern level, who are not truly representative of those pursuing speciality training. Finally, no responses were obtained from those in specialties such as anaesthesia or obstetrics, specialties which are also skills based and would have made for interesting comparison.

## Data Availability

All data can be made available in Excel format from the authors upon request.
